# Estimating the malaria transmission of *Plasmodium vivax* based on serodiagnosis

**DOI:** 10.1186/1475-2875-11-257

**Published:** 2012-08-01

**Authors:** Jung-Yeon Kim, Hyung-Hwan Kim, Byoung-Kuk Na, Yeon-Joo Kim, Youngjoo Sohn, Hyuck Kim, Tong-Soo Kim, Hyeong-Woo Lee

**Affiliations:** 1 Division of Malaria and Parasitic Diseases, Korea Centers for Disease Control and Prevention, Osong 363-951, Republic of Korea; 2 Vascular Medicine Research Unit, Brigham and Women's Hospital, Harvard Medical School, Cambridge, MA 02139, USA; 3 Department of Parasitology and Institute of Health Sciences, Gyeongsang National University School of Medicine, Jinju 660-751, Republic of Korea; 4 Department of Anatomy, College of Oriental Medicine, Institue of Oriental Medicine, Kyung Hee University, Hoegi-dong, Dongdaemun-gu, Seoul 130-701, Republic of Korea; 5 Department of Parasitology, Inha University School of Medicine, Incheon 400-712, Republic of Korea; 6 International Research Center for Bioscience and Biotechnology, Jungwon University, Goesan 367-805, Republic of Korea; 7 Department of Pathology, University of Florida, J-566, 1275 Center Drive, Gainesville, FL 32610, USA

## Abstract

**Background:**

*Plasmodium vivax* re-emerged in 1993 and has now become a major public health problem during the summer season in South Korea. The aim of this study was to interpret and understand the meaning of seroepidemiological studies for developing the best malaria control programme in South Korea.

**Methods:**

Blood samples were collected in Gimpo city, Paju city, Yeoncheon County, Cheorwon County and Goseong County of high risk area in South Korea. Microscopy was performed to identify patients infected with *P. vivax*. Antibody detection for *P. vivax* was performed using indirect fluorescent antibody test (IFAT).

**Results:**

A total of 1,574 blood samples was collected from participants in the study areas and evaluated against three parameters: IFAT positive rate, annual antibody positive index (AAPI), and annual parasite index (API). The IFAT positive rate was 7.24% (n = 114). Of the five study areas, Gimpo had the highest IFAT positive rate (13.68%) and AAPI (4.63). Yeongcheon had the highest API in 2005 (2.06) while Gimpo had the highest API in 2006 (5.00). No correlation was observed between any of the three parameters and study sites' distance from the demilitarized zone (DMZ).

**Conclusions:**

These results showed that *P. vivax* antibody levels could provide useful information about the prevalence of malaria in endemic areas. Furthermore, AAPI results for each year showed a closer relationship to API the following year than the API of the same year and thus could be helpful in predicting malaria transmission risks.

## Background

*Plasmodium vivax* is the causative agent of relapsing benign tertian human malaria and is the second-most important human malaria that annually afflicts several hundred million people. The disease is a major public health problem and has socio-economic ramifications for many temperate and tropical countries [1]. While vivax malaria has been reported throughout the Korean peninsula for several centuries, it was not until 1913 that the first scientific document was published. At that time, malaria occurred throughout the country without recognizable geographical differences [2]. The incidence of vivax malaria decreased rapidly as a result of economic improvement following the Korean War, a national malaria eradication programme, and assistance from the World Health Organization (WHO) [3,4]. As the last two sporadic cases detected in the 1980s were believed to be the result of latent malaria parasites transmitted the previous year [5], vivax malaria was reported to have been eradicated in South Korea by the late 1970s [6]. In 1993, a South Korean army soldier serving in northern Gyeonggi Province, with no travel history, was diagnosed with vivax malaria [7]. Subsequently, Cho *et al.* reported two civilian patients infected with vivax malaria [8]. By 2005, a total of 21,419 indigenous vivax malaria cases had been confirmed in South Korea, and a total of at least 937,634 vivax malaria cases had been reported from the entire Korean peninsula, both South and North Korea. The number of vivax malaria cases peaked in South Korea in 2000 with 8.9 cases/100,000, followed by a sharp decline of approximately 26-40% per annum to 1.8 and 2.9 cases/100,000 in 2004 and 2005, respectively [9]. The highest malaria cases centred around Paju, Yeoncheon, Cheorwon, Gimpo, Ganghwa, Goyang, and Dongducheon near the demilitarized zone (DMZ) separating North and South Korea. Following the re-emergence of malaria, subsequent high indigenous transmission rates and population movement caused great concern because of the increased geographical expansion potential [10].

Serological data obtained by an indirect fluorescent antibody test (IFAT) may provide useful for levels of malaria endemicity, as well as the time period of infection [11]. Of blood samples from 845 participants, who were residents of Gimpo from November to December 1998, 24 were positive for malaria antibodies by IFAT. Four seropositive participants (16.7%) developed malaria the following year. In 1999, 125 of 5,797 participants from the same area were seropositive by IFAT of which 12.8% (16/125) were positive for malaria parasites by polymerase chain reaction (PCR). Serological surveys have provided valuable epidemiological information, especially in areas with low level of endemicity [12,13]. The rate of parasitaemia is the classical method for measuring the endemicity of malaria, however, the incidence of parasitaemia alone fails to provide an adequate description of the occurrence of malaria in a population. Therefore, the incidence of malaria is low, the application of IFAT could be used to more accurately reflect the malaria situation in a particular area [14,15].

In this study, antibody-positive rates using IFAT were obtained from malaria high-risk areas near the DMZ and the results compared with the malaria prevalence in those areas during the year of the survey and the following year.

## Methods

### Study areas and blood sample collection

**Figure 1:**
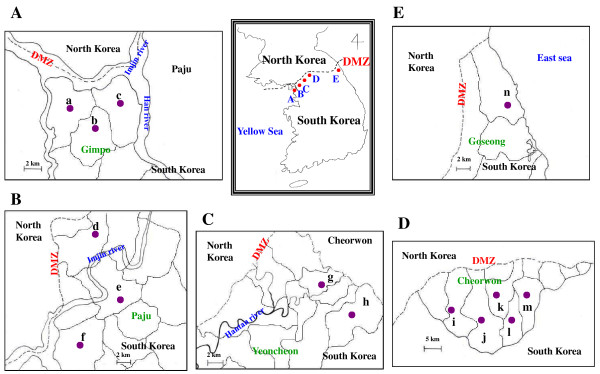
**Study areas.** (**A**) Gimpo; (**B**) Paju; (**C**) Yeoncheon; (**D**) Cheorwon; (**C**) Goseong. **a**, Wolgot village; **b**, Tongjin village; **c**, Haseong village; **d**, Gunnae village; **e**, Munsan village; **f**, Tanhyun village; **g**, Yeoncheon village; **h**, Cheongsan village; **i**, Dongsong village; **j**, Galmal village; **k**, Gimhwa village; **l**, Seo village; **m**, Geunnam village; **n**, Hyeonnae village.

The study sites were within 20 km of the DMZ and are shown in Figure 1. The study was conducted in Gimpo (Figure 1A), Paju (Figure 1B), and Yeoncheon (Figure 1C) of Gyeonggi Province, and Cheorwon (Figure 1D) and Goseong (Figure 1E) of Gangwon Province, South Korea, from late October to mid-December 2005. Blood samples were collected from participants residing in 35 villages in three cities located in Gyeonggi Province, and nine villages in two cities in Gangwon Province. A total of 1,574 blood samples (1.43%) were collected from 110,424 inhabitants of the study areas in 2005. All participants were volunteers enrolled by providing verbal informed consent. Blood samples were collected randomly from volunteers and ruled out those who had previously been infected with malaria. Three ml of blood was collected from each individual and thin and thick blood smears prepared for microscopic examination (magnification 7 x 100). The blood samples were transferred to the Korean National Institute of Health (KNIH), Korea Centers for Disease Control and Prevention (KCDC), where blood and sera were separated and stored at −20°C for future antibody analysis. The study protocol was reviewed and approved by the KNIH Human Ethics Committee.

### Indirect fluorescent antibody test

To test for antibodies against malaria, IFAT was performed with the whole blood infected with *P. vivax *[16-18]. Briefly, malaria parasite infected blood was collected from patients. Plasma and white blood cells were removed and red blood cells suspended in phosphate buffered saline (PBS, pH 7.2). Samples were centrifuged for 5 min at 2,500 rpm. The supernatant was discarded and the cells were resuspended in fresh PBS. This wash step was repeated three more times, and then an appropriate amount of PBS was added. Red blood cells were mounted in each well of Teflon-coated slides, dried for 12 hr at room temperature, and then stored at −70°C. To determine the antibody titres against *P. vivax* of each individual, the antigen slides were fixed in pre-cooled acetone (−20°C) for 10 min, washed with PBS, and then 20 μl of diluted sera, 1:32 to 1:8,192 (vol/vol), was added to each well. Positive and negative controls were also spotted onto each slide, and the slides were incubated in a humidified chamber for 30 min at 37°C. The reactions were quenched by washing the reacted sera with PBS for 6 min, and then the samples were dried at room temperature. Diluted FITC conjugated anti-human IgG (Sigma, 1:32 vol/vol in PBS) was added to each well and incubated and washed as described above. Several drops of buffered glycerol were added to the samples, coverslips applied and then examined under a fluorescence microscope at 400x.

### Calculation of annual parasite index (API)

API was calculated as the number of malaria-positive patients per 1,000 inhabitants at each of the study sites, API = (number of positive slides/total number of slides) × 1000 [19].

### Calculation of annual antibody positive index (AAPI)

AAPI was calculated as the number of malaria antibody positive participants per 1,000 inhabitants for each of the study sites, AAPI = (number of antibody positive slides/total number of slides) × 1000.

### Data analysis

The antibody-positive individuals who had been patients in previous years were excluded during data analysis. The relationship of distance from the DMZ as related to IFAT positive rate, API and AAPI were analysed by Pearson’s chi-squared test. The relationship between AAPI, API, and IFAT positive rate for each village was analysed by two-way ANOVA. Data analyses were performed using GraphPad (GraphPad Software, Inc., La Jolla, CA, USA).

## Results

### IFAT positive rate

The criteria for a positive IFAT result were established in a previous experiment. Briefly, when 86 individuals and 58 vivax malaria patients were tested, normal individuals had a serum dilution under 1:16 (range from 0 to 1:16), and the 58 vivax malaria patients had a serum dilution over 1:256 (range from 1:256 to 1:4,096). Therefore, a positive antibody response was defined against vivax malaria as a serum dilution above (≥)1:32. The specificity of IFAT was 100% and sensitivity was 97% [20]. A total of 114 samples from 1,574 participants (7.24%) that were identified as negative by microscopic examination were positive by IFAT. A total 17 positive samples exhibited IFAT scores of ≥1:256, which was the criterion used to identify malaria patients. Gimpo had the highest positive rate at 13.68% (52/380) followed by Cheorwon (7.01%, 27/385), Paju (5.51%, 26/472), Yeoncheon (3.32%, 7/211), and Goseong (1.59%, 2/126) (Additional file 1). Tongjin village, located in the upper-middle area of Gimpo, had the highest positive rate at 17.65% (9/51) followed by Wolgot village (14.63%, 24/164) in the north-west part of the city, and Haseong village (11.52%, 19/165) in the north-east (Additional file 2, Figure 1A). Gunnae village, located adjacent to the DMZ in Paju, had the highest positive rate of 8.89% (8/90) followed by Munsan village (7.26%, 13/179) in the upper-middle of the city, and Tanhyun village (2.46%, 5/203) in the north-west (Additional file 3, Figure 1B). Cheongsan village, located far south of Yeoncheon, had the highest positive rate at 5.41% (2/37) followed by Yeoncheon village (2.87%, 5/174) located in the middle of Yeoncheon (Additional file 4, Figure 1C). Galmal village, located in the south-west of Cheorwon, had the highest positive rate at 11.48% (7/61) followed by Seo village (9.52%, 4/42) in the south-east of the city, Geunnam village (9.09%, 5/55) and Gimhwa village (6.67%, 2/30) in the north-east, and Dongsong village (4.57%, 9/197) in the west of the city (Additional file 5, Figure 1D). Hyeonnae village, located in the north part of Goseong, had a positive rate of 1.59% (2/126) (Additional file 6, Figure 1E).

### Comparison of the annual antibody positive index (AAPI)

The AAPI method was designed to normalize the IFAT positive rates. Total AAPI was 1.03 for study sites within Gimpo showing the highest AAPI (4.63) followed by Paju (0.64), Cheorwon (0.62), Yeoncheon (0.60), and Goseong (0.57) (Additional file 1). Wolgot village showed the highest AAPI in Gimpo (12.04) followed by Haseong village (7.30), and Tongjin village (1.36) (Additional file 2, Figure 1A). Gunnae village had the highest AAPI in Paju (11.68) followed by Munsan village (0.46) and Tanhyun village (0.42) (Additional file 3, Figure 1B). Yeoncheon village (0.71) showed a higher AAPI than Cheongsan village (0.43) in Yeoncheon (Additional file 4, Figure 1C). Geunnam village showed the highest AAPI in Cheorwon (1.90) followed by Gimhwa village (0.58), Seo village (0.58), Dongsong village (0.54), and Galmal village (0.51) (Additional file 5, Figure 1D). The AAPI of Hyeonnae village in Goseong was 0.57 (Additional file 6, Figure 1E).

### Comparison of annual parasite index (API) results from 2005 and 2006

Total API for 2005 was 1.24 and 1.89 in 2006. The highest API was detected in Yeoncheon (2.06) followed by Cheorwon (1.45), Gimpo (1.07), and Paju (0.94). There were no malaria patients in Goseong in 2005. The highest API was detected in Gimpo (5.00) followed by Paju (1.80), Cheorwon (1.45), Yeoncheon (1.03), and Goseong (0.70). The AAPI results from 2005 indicated a closer relationship to the API of 2006 (*P* = 0.0003) than to the API of 2005 (*P* = 0.7436) (Additional file 1).

In 2005, the highest API in Gimpo was observed in Wolgot village (2.01), followed by Tongjin village (1.21) and Haseong village (0.00). In 2006, Wolgot village showed the highest API followed by Haseong village (6.54) and Tongjin village (2.41) (Figure 1A).

Similarly, a closer relationship was observed between the AAPI of 2005 and the API of 2006 (*P* = 0.0097) than between the AAPI of 2005 and the API of 2005 (*P* = 0.4388) (Additional file 2). In 2005, the highest API in Munsan village was detected in Paju (1.14) followed by Tanhyun village (0.51) and Gunnae village (0.00). In 2006, the highest API was in Gunnae village (5.84) followed by Munsan village (1.82) and Tanhyun village (1.52) (Additional file 3, Figure 1B). In Yeoncheon, Yeoncheon village (2005; 3.41, 2006; 1.14) maintained a higher positive API than Cheongsan village (2005: 0.00; 2006: 0.87). Here, the 2005 AAPI showed a closer relationship to the API of 2006 (*P* = 0.0116) than the API of 2005 (*P* = 0.4478) (Additional file 4, Figure 1C). For Cheorwon in 2005, the highest API was detected in Gimhwa village (2.33) followed by Geunnam village (1.90), Dongsong village (1.66), Galmal village (1.54), and Seo village (0.14). In 2006, Gimhwa village had the highest API (2.63) followed by Geunnam village (1.90), Dongsong village (1.37), Seo village (1.30), and Galmal village (1.25) (Additional file 5, Figure 1D). The API in Hyeonnae village in Goseong increased from 2005 (0.00) to 2006 (0.70) (Additional file 6, Figure 1E).

### Comparison between each parameter and distance from the DMZ

Vivax malaria in South Korea is characterized as border malaria [10,21]. It was tried to confirm this hypothesis by investigating whether the distance from the DMZ to each study sites influenced the IFAT positive rate, AAPI, or API in 2005 and 2006. In 2005, in the study areas of Gimpo, the positive rates for IFAT (r^2^ = 0.9607, *P* = 0.1271) and API (r^2^ = 0.1897, *P* = 0.7131) increased with distance from DMZ, and in 2006, AAPI (r^2^ = 0.4931, *P* = 0.5044) and API (r^2^ = 0.9411, *P* = 0.1560) decreased with distance. However, none of the parameters was significant (*P* > 0.05). In 2006, the study areas of Paju, the positive rates for for IFAT (r^2^ = 0.0497, *P* = 0.8569), AAPI (r^2^ = 0.7473, *P* = 0.3353) and API (r^2^ = 0.6943, *P* = 0.3729) decreased with distance from the DMZ. Only 2005 results showed significant increase of API with proximity to the DMZ (r^2^ = 0.9963, *P* = 0.0386) (*P* < 0.05). AAPI and API (2005 and 2006) decreased with distance from the DMZ in the study areas of Yeoncheon. Only the IFAT positive rate increased with proximity to the DMZ. 2005 AAPI (r^2^ = 0.4539, *P* = 0.2124), 2005 API (r^2^ = 0.4068, *P* = 0.2469), and the 2006 API rates (r^2^ = 0.5556, *P* = 0.1482) decreased with distance from the DMZ in the study areas of Cheorwon. Only positive rates of IFAT (r^2^ = 0.2542, *P* = 0.3864) increased, but the data were not significance (*P* > 0.05).

## Discussion

The highest prevalence of malaria in South Korea was observed for Paju and Yeoncheon, in Gyeonggi Province, both of which are located within 10–15 km of the southern border of the DMZ [10,21]. Therefore, it was tried to determine the malaria transmission pattern in the malaria-prevalent areas: Gimpo, Paju, Yeoncheon, Cheorwon, and Goseong.

Based on the data, Tongjin village in Gimpo may be dominated by locally transmitted malaria, because, in 2005, the IFAT positive (17.65%) and API (1.21) indices for that village were much higher than those for Wolgot village (5.70 km) and Haseong village (5.00 km), which are closer to the DMZ (8.00 km). However, Wolgot village had relatively high values for all three parameters and was closest to the DMZ, suggesting that the malaria transmission increased near the border. One of the main rice fields is located in Gimpo, South Korea where there are many places for *Anopheles* to breed, which suggests the main reason for the high malaria prevalence (Additional file 2). Gunnae village of Paju showed a higher IFAT positive rate and higher AAPI than Musan village or Tanhyun village, but there were no malaria patients reported in 2005 (API = 0.00). However, the API was 5.84 in 2006. This suggests that antibody-positive rates might be useful for predicting the malaria transmission in subsequent years. A new approach to interpreting AAPI results would be helpful to more accurately estimate community immunity in order to predict malaria transmission in epidemic areas (Additional file 3). In 2005, the AAPI (0.71) and API (3.41) of Yeoncheon village, located close to DMZ, exhibited higher values than did Cheongsan village in Yeoncheon, suggesting that Yeoncheon may have been directly affected by mosquitoes along the border with North Korea (Additional file 4). Similarly, the AAPI and API of villages in Cheorwon decreased as distance increased from the DMZ. Geunnam village, located close to the DMZ, showed the highest value of AAPI (1.90) and second highest API (2005: 1.90; 2006: 1.90) among the villages surveyed in Cheorwon. In 2005, AAPI for the other areas was between 0.51 and 0.58, but the positive rate observed in Galmal village, located far from the DMZ (18.18 km), exhibited the highest positive rate (11.48%) and an average value of API (2005: 1.54; 2006: 1.25). Thus, Galmal village exhibited the characteristics of local malaria transmission. Gimhwa village exhibited the highest values for API (2005: 2.33; 2006: 2.63) and second-highest value for AAPI, and is located close to the DMZ (Additional file 5). When it was considered the relationship between AAPI and API, AAPI results showed significantly greater correlation with API in 2006 (*P* = 0.0003) than in 2005 (*P* = 0.7436). From this, it was concluded that the AAPI results on any given year may indicate the malaria transmission pattern for the following year. Because it would be helpful to be able to predict the malaria transmission in following years, this hypothesis should be tested for the purpose of developing diagnostic tools to aid in eradicating malaria in Korea. AAPI results may correlate with the long incubation period of malaria in patients, when it is taken into consideration the time of blood collection, as adult mosquitoes are not present or in hibernation during the cold winter season. In humans, the malaria parasites are dormant in the liver as hypnozoites, with development usually initiated during the following mosquito season. Going forward, it must be determined the AAPI before malaria transmission occurs to determine precisely what this index offers.

IFAT was applied in this study because serological data provide useful evidence regarding the extent and degree of malaria endemicity, and reflect the period of infection [11], particularly in areas with low endemicity [13]. Additionally, sensitivity and specificity of IFAT analysis are much higher than enzyme linked immunosorbent assay (ELISA) of merozoite surface protein-1 (MSP-1) and circumsporozoite surface protein (CSP) in previous data [20]. The parasitaemia rate is the classical method for measuring malaria endemicity, but incidence of parasitaemia alone cannot provide a complete and adequate description of malaria infections among affected populations. When the incidence of malaria is low, mass blood surveys based on microscopic examination do not yield results commensurate with the work involved [14]. Therefore, the application of IFAT could be utilized to more accurately reflect the malaria situation in a given population [15].

If non-latent cases are observed in early June, sporozoites infection from mosquitoes to humans would have occurred in mid-May of the same year. During April, mosquitoes are usually infected with gametocytes by blood meal from latent patients and would have to have developed sporozoites in the salivary gland by mid-May to be infective to human beings. Malaria cases observed in May are largely due to latent malaria cases from the previous year [22].

The most abundant anopheline mosquitoes captured in the high-risk areas for malaria near the DMZ in northern Gyeonggi province were *Anopheles sinensis* (63.3%), *Anopheles kleini* (24.7%), and *Anopheles pullus* (8.7%) [23]. The incidence of malaria peaks in August after the rainy season and declines to baseline by mid-October. Blood collections were conducted between late October and mid-December when the active adult anopheline population has disappeared. It was conducted the same experimental design from 1996 to 1998, during the early stage of re-emergence of vivax malaria in South Korea, and the IFAT-determined antibody-positive rates in the study areas of Paju, Yeoncheon, and Cheorwon were highly influenced by the proximity to the DMZ. As some of the study areas far from the DMZ showed high positive rates for IFAT, AAPI, and API in 2005 and 2006, it appears that the pattern of vivax malaria transmission has changed and is no longer solely affected by border conditions. In other words, the causative source of malaria transmission is now located inside South Korea.

## Conclusions

An interesting finding of this study was that antibody detection using IFAT may provide useful information regarding malaria prevalence in certain areas and individuals. These data may assist in identifying the areas at increased risk, and which require a more or less intensive surveillance the following years. Additionally, antibody detection helps to find asymptomatic patients who play an important role in malaria transmission.

## Competing interests

The authors declare that they have no competing interests.

## Authors’ contributions

TSK, JYK, and HWL conceived and designed the study as well as contributed to the execution of the research. HWL wrote the manuscript. HHK, SY, and HK contributed statistical analysis. TSK, BKN, JYK, and HWL collected the blood samples in the field. HWL and YJK performed IFAT. All authors have read and approved the final manuscript.

## Supplementary Material

Additional file 1: Positive rate and distribution of fluorescent antibody responses of sera by surveyed area. Click here for file

Additional file 2: Positive rate of fluorescent antibody responses of sera in Gimpo surveyed area. Click here for file

Additional file 3: Positive rate of fluorescent antibody responses of sera in Paju surveyed area. Click here for file

Additional file 4: Positive rate of fluorescent antibody responses of sera in Yeoncheon surveyed area. Click here for file

Additional file 5: Positive rate of fluorescent antibody responses of sera in Cheorwon surveyed area. Click here for file

Additional file 6: Positive rate of fluorescent antibody responses of sera in Goseong surveyed area. Click here for file

## References

[B1] Mendis K Sina BJ Marchesini P Carter R The neglected burden of Plasmodium vivax malaria Am J Trop Med Hyg 2001 64 97 106 11425182 10.4269/ajtmh.2001.64.97

[B2] Hasegawa Malaria in Korea Chosen Igakkai Zasshi 1913 4 53 69

[B3] National Malaria Eradication Service, Ministry of Health and Social Affairs, ROK Malaria pre-eradication programme in Korea, 1961–1965. Progress report 1966 44 70

[B4] Paik YH Rhee HI Shim JC Malaria in Korea Jpn J Exp Med 1988 58 55 66 3045377

[B5] Soh CT Lee KT Im KI Min DY Ahn MH Kim JJ Yong TS Current status of malaria in Korea Yonsei Rep Trop Med 1985 16 11 18

[B6] World Health Organization Synopsis of the world malaria situation, 1979 Weekly Epidemiol Rec 1981 56 145 149

[B7] Chai IH Lim GI Yoon SN Oh WI Kim SJ Chai JY [Occurrence of tertian malaria in a male patient who has never been abroad](in Korean) Korean J Parasitol 1994 32 195 200 10.3347/kjp.1994.32.3.195 7953245

[B8] Cho SY Kong Y Park SM Lee JS Lim YA Chae SL Kho WG Lee JS Shim JC Shin HK Two vivax malaria cases detected in Korea Korean J Parasitol 1994 32 281 284 10.3347/kjp.1994.32.4.281 7834248

[B9] Han ET Lee DH Park KD Seok WS Kim YS Tsuboi T Shin EH Chai JY Reemerging vivax malaria: changing patterns of annual incidence and control programs in the Republic of Korea Korean J Parasitol 2006 44 285 294 10.3347/kjp.2006.44.4.285 17170570 PMC2559126

[B10] Lee JS Kho WG Lee HW Seo M Lee WJ Current status of vivax malaria among civilians in Korea Korean J Parasitol 1998 36 241 248 10.3347/kjp.1998.36.4.241 9868889 PMC2732963

[B11] Jeffery GM McWilson W Collins WE Lobel H Application of the indirect fluorescent antibody method in a study of malaria endemicity in Mato Grosso, Brazil Am J Trop Med Hyg 1975 24 402 411 1098491 10.4269/ajtmh.1975.24.402

[B12] Lee WJ Kim HH Hwan SM Park MY Kim NR Cho SH In TS Kim JY Sattabongkot J Sohn YJ Lee JK Lee HW Detection of an antibody against Plasmodium vivax in residents of Gimpo-si, South Korea, using an indirect fluorescent antibody test Malar J 2011 10 19 10.1186/1475-2875-10-19 21281481 PMC3042984

[B13] Cerutti C Boulos M Coutinho AF Hatab MC Falqueto A Rezende HR Duarte AM Collins W Malafronte RS Epidemiologic aspects of the malaria transmission cycle in an area of very low incidence in Brazil Malar J 2007 6 33 10.1186/1475-2875-6-33 17371598 PMC1839104

[B14] Collins EC Skinner JC The indirect fluorescent antibody test for malaria Am J Trop Med Hyg 1972 21 690 695 4627546 10.4269/ajtmh.1972.21.690

[B15] Wang DQ Tang LH Gu ZC Zheng X Yang MN Application of the indirect fluorescent antibody assay in the study of malaria infection in the Yangtze River Three Gorges Reservoir, China Malar J 2009 8 199 10.1186/1475-2875-8-199 19678949 PMC3224913

[B16] Sulzer AJ Wilson M Hall EC Indirect fluorescent-antibody tests for parasitic diseases. V. An evaluation of a thick-smear antigen in the IFA test for malaria antibodies Am J Trop Med Hyg 1969 18 199 205 4888028

[B17] Collins WE Skinner JC The indirect fluorescent antibody test for malaria AmJTrop Med Hyg 1972 21 690 695 10.4269/ajtmh.1972.21.690 4627546

[B18] Collins WE Warren M Skinner JC Fredericks HJ Studies on the relationship between fluorescent antibody response and ecology of malaria in Malaysia Bull World Health Organ 1968 39 451 463 4882987 PMC2554424

[B19] Rodriques Eda C Lopes Neto D Malaria control in an Amazon municipality Rev Lat Am Enfermagem 2011 19 1297 1305 10.1590/S0104-11692011000600004 22249662

[B20] Lee HW Lee JS Lee WJ Cho SH Lee HS [The evaluation of recombinant circumsporozoite protein in malaria diagnosis] (in Korean) Korean J Microbiol 2000 36 142 149

[B21] Kho WG Jang JY Hong ST Lee HW Lee WJ Lee JS Border malaria characters of reemerging vivax malaria in the Republic of Korea Korean J Parasitol 1999 37 71 76 10.3347/kjp.1999.37.2.71 10388264 PMC2733059

[B22] Park JW Klein TA Lee HC Pacha LA Ryu SH Yeom JS Moon SH Kim TS Chai JY Oh MD Choe KW Vivax malaria: a continuing health threat to the Republic of Korea Am J Trop Med Hyg 2003 69 159 167 13677372

[B23] Lee WJ Klein TA Kim HC Choi YM Yoon SH Chang KS Chong ST Lee IY Kohes JW Jacobs JS Sattabongkot J Park JS Anopheles kleini, Anopheles pullus, and Anopheles sinensis: potential vectors of Plasmodium vivax in the Republic of Korea J Med Entomol 2007 44 1086 1090 10.1603/0022-2585(2007)44[1086:AKAPAA]2.0.CO;2 18047210

