# Supplementary material for: Estimating the malaria transmission of Plasmodium vivax based on serodiagnosis
Source: Malar J. 2012 Aug 1;11:257. doi: 10.1186/1475-2875-11-257 (PMC3470937; doi:10.1186/1475-2875-11-257)
Supplement: Additional file 1: — Positive rate and distribution of fluorescent antibody responses of sera by surveyed area. [file 1475-2875-11-257-S1.ppt]

## Slide 1
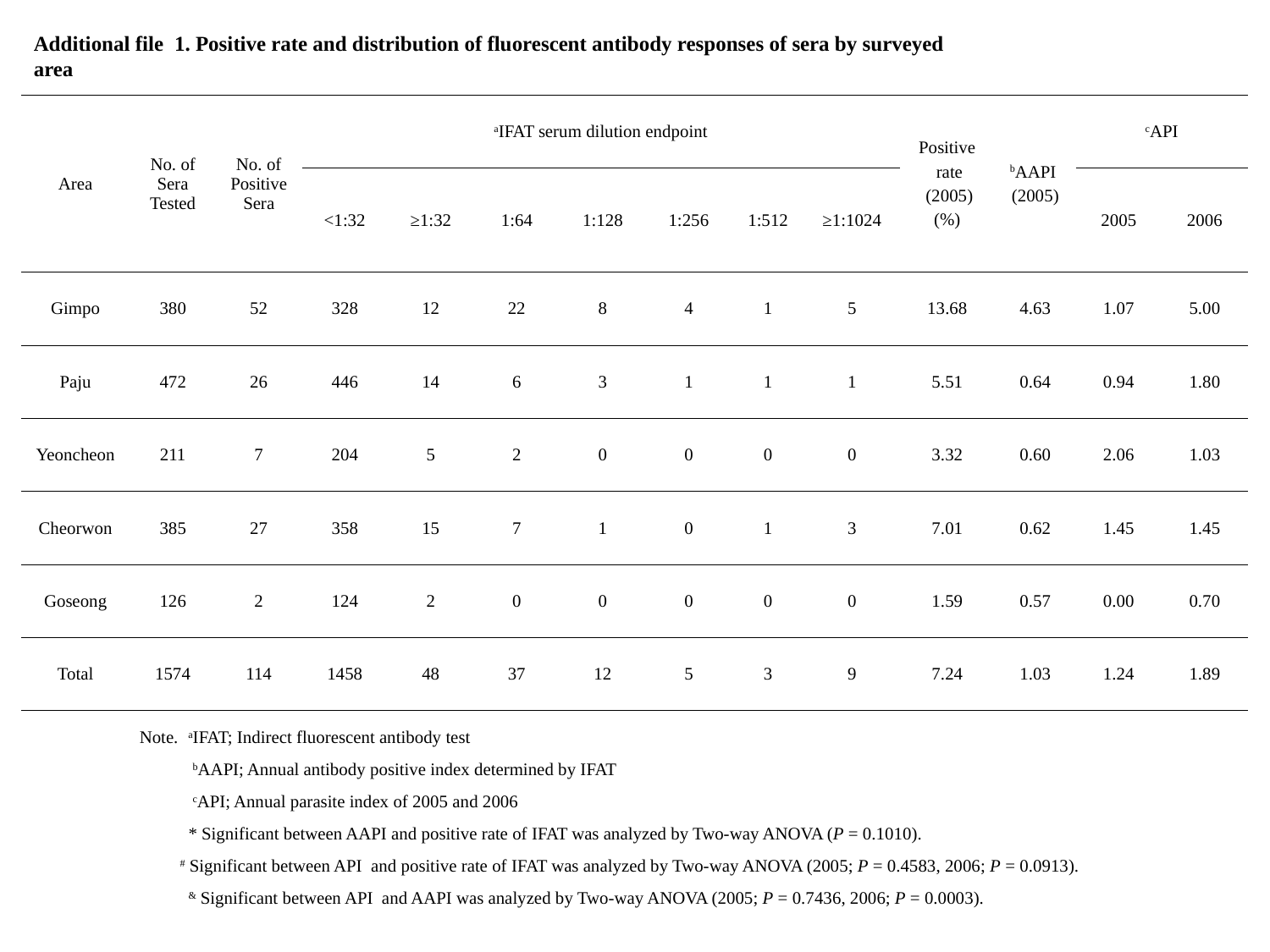

Additional file 1. Positive rate and distribution of fluorescent antibody responses of sera by surveyed area
| Area | No. of Sera Tested | No. of Positive Sera | aIFAT serum dilution endpoint | | | | | | | Positive rate (2005) (%) | bAAPI (2005) | cAPI | |
| --- | --- | --- | --- | --- | --- | --- | --- | --- | --- | --- | --- | --- | --- |
| | | | <1:32 | ≥1:32 | 1:64 | 1:128 | 1:256 | 1:512 | ≥1:1024 | | | 2005 | 2006 |
| Gimpo | 380 | 52 | 328 | 12 | 22 | 8 | 4 | 1 | 5 | 13.68 | 4.63 | 1.07 | 5.00 |
| Paju | 472 | 26 | 446 | 14 | 6 | 3 | 1 | 1 | 1 | 5.51 | 0.64 | 0.94 | 1.80 |
| Yeoncheon | 211 | 7 | 204 | 5 | 2 | 0 | 0 | 0 | 0 | 3.32 | 0.60 | 2.06 | 1.03 |
| Cheorwon | 385 | 27 | 358 | 15 | 7 | 1 | 0 | 1 | 3 | 7.01 | 0.62 | 1.45 | 1.45 |
| Goseong | 126 | 2 | 124 | 2 | 0 | 0 | 0 | 0 | 0 | 1.59 | 0.57 | 0.00 | 0.70 |
| Total | 1574 | 114 | 1458 | 48 | 37 | 12 | 5 | 3 | 9 | 7.24 | 1.03 | 1.24 | 1.89 |
Note. aIFAT; Indirect fluorescent antibody test
 bAAPI; Annual antibody positive index determined by IFAT
 cAPI; Annual parasite index of 2005 and 2006
 * Significant between AAPI and positive rate of IFAT was analyzed by Two-way ANOVA (P = 0.1010).
 # Significant between API and positive rate of IFAT was analyzed by Two-way ANOVA (2005; P = 0.4583, 2006; P = 0.0913).
 & Significant between API and AAPI was analyzed by Two-way ANOVA (2005; P = 0.7436, 2006; P = 0.0003).
