# Supplementary material for: Estimating the malaria transmission of Plasmodium vivax based on serodiagnosis
Source: Malar J. 2012 Aug 1;11:257. doi: 10.1186/1475-2875-11-257 (PMC3470937; doi:10.1186/1475-2875-11-257)
Supplement: Additional file 2: — Positive rate of fluorescent antibody responses of sera in Gimpo surveyed area. [file 1475-2875-11-257-S2.ppt]

## Slide 1
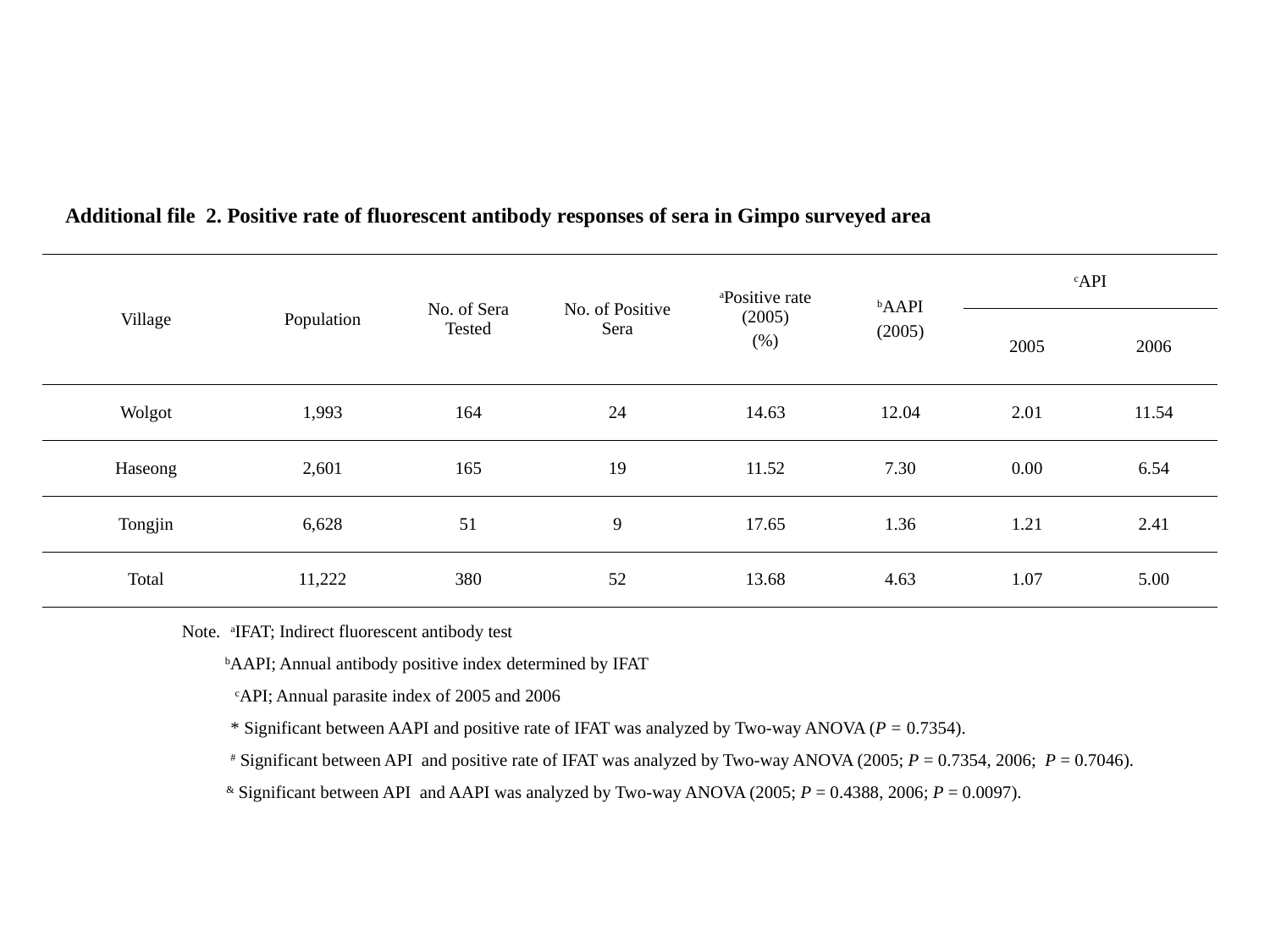

Additional file 2. Positive rate of fluorescent antibody responses of sera in Gimpo surveyed area
| Village | Population | No. of Sera Tested | No. of Positive Sera | aPositive rate (2005) (%) | bAAPI (2005) | cAPI | |
| --- | --- | --- | --- | --- | --- | --- | --- |
| | | | | | | 2005 | 2006 |
| Wolgot | 1,993 | 164 | 24 | 14.63 | 12.04 | 2.01 | 11.54 |
| Haseong | 2,601 | 165 | 19 | 11.52 | 7.30 | 0.00 | 6.54 |
| Tongjin | 6,628 | 51 | 9 | 17.65 | 1.36 | 1.21 | 2.41 |
| Total | 11,222 | 380 | 52 | 13.68 | 4.63 | 1.07 | 5.00 |
Note. aIFAT; Indirect fluorescent antibody test
 bAAPI; Annual antibody positive index determined by IFAT
 cAPI; Annual parasite index of 2005 and 2006
 * Significant between AAPI and positive rate of IFAT was analyzed by Two-way ANOVA (P = 0.7354).
 # Significant between API and positive rate of IFAT was analyzed by Two-way ANOVA (2005; P = 0.7354, 2006; P = 0.7046).
 & Significant between API and AAPI was analyzed by Two-way ANOVA (2005; P = 0.4388, 2006; P = 0.0097).
