# Supplementary material for: Estimating the malaria transmission of Plasmodium vivax based on serodiagnosis
Source: Malar J. 2012 Aug 1;11:257. doi: 10.1186/1475-2875-11-257 (PMC3470937; doi:10.1186/1475-2875-11-257)
Supplement: Additional file 3: — Positive rate of fluorescent antibody responses of sera in Paju surveyed area. [file 1475-2875-11-257-S3.ppt]

## Slide 1
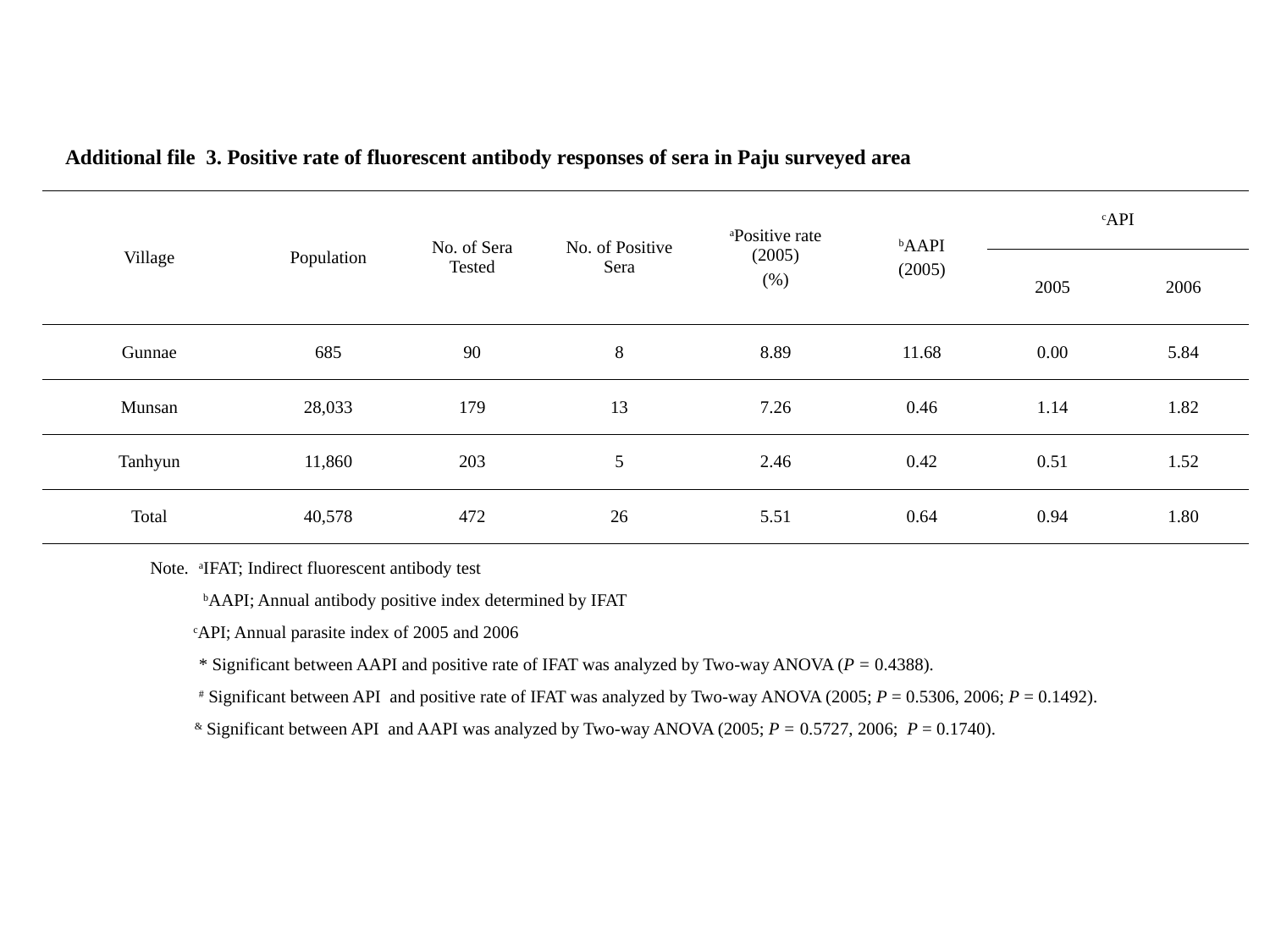

Additional file 3. Positive rate of fluorescent antibody responses of sera in Paju surveyed area
| Village | Population | No. of Sera Tested | No. of Positive Sera | aPositive rate (2005) (%) | bAAPI (2005) | cAPI | |
| --- | --- | --- | --- | --- | --- | --- | --- |
| | | | | | | 2005 | 2006 |
| Gunnae | 685 | 90 | 8 | 8.89 | 11.68 | 0.00 | 5.84 |
| Munsan | 28,033 | 179 | 13 | 7.26 | 0.46 | 1.14 | 1.82 |
| Tanhyun | 11,860 | 203 | 5 | 2.46 | 0.42 | 0.51 | 1.52 |
| Total | 40,578 | 472 | 26 | 5.51 | 0.64 | 0.94 | 1.80 |
Note. aIFAT; Indirect fluorescent antibody test
 bAAPI; Annual antibody positive index determined by IFAT
 cAPI; Annual parasite index of 2005 and 2006
 * Significant between AAPI and positive rate of IFAT was analyzed by Two-way ANOVA (P = 0.4388).
 # Significant between API and positive rate of IFAT was analyzed by Two-way ANOVA (2005; P = 0.5306, 2006; P = 0.1492).
 & Significant between API and AAPI was analyzed by Two-way ANOVA (2005; P = 0.5727, 2006; P = 0.1740).
