# Supplementary material for: Estimating the malaria transmission of Plasmodium vivax based on serodiagnosis
Source: Malar J. 2012 Aug 1;11:257. doi: 10.1186/1475-2875-11-257 (PMC3470937; doi:10.1186/1475-2875-11-257)
Supplement: Additional file 4: — Positive rate of fluorescent antibody responses of sera in Yeoncheon surveyed area. [file 1475-2875-11-257-S4.ppt]

## Slide 1
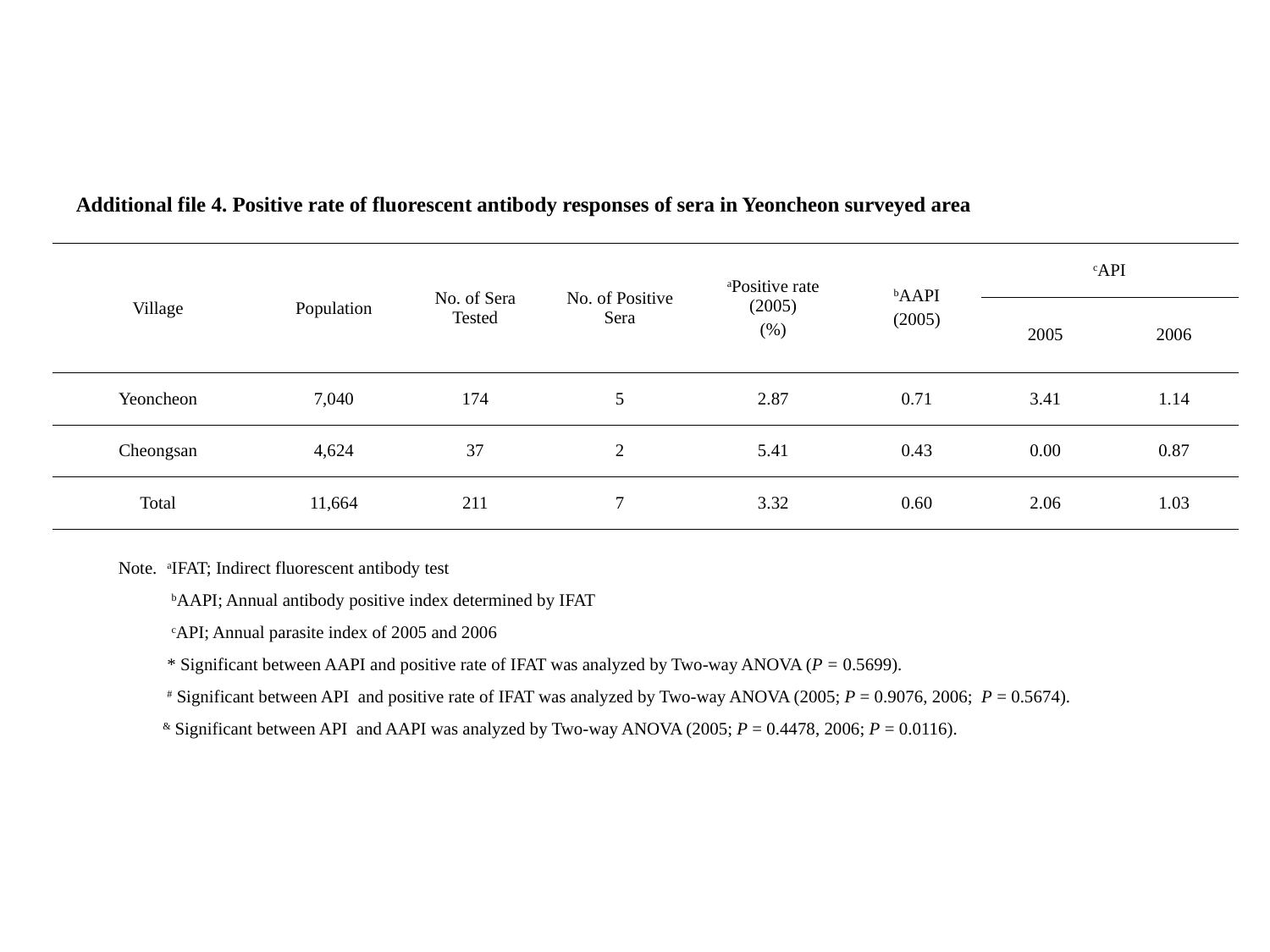

Additional file 4. Positive rate of fluorescent antibody responses of sera in Yeoncheon surveyed area
| Village | Population | No. of Sera Tested | No. of Positive Sera | aPositive rate (2005) (%) | bAAPI (2005) | cAPI | |
| --- | --- | --- | --- | --- | --- | --- | --- |
| | | | | | | 2005 | 2006 |
| Yeoncheon | 7,040 | 174 | 5 | 2.87 | 0.71 | 3.41 | 1.14 |
| Cheongsan | 4,624 | 37 | 2 | 5.41 | 0.43 | 0.00 | 0.87 |
| Total | 11,664 | 211 | 7 | 3.32 | 0.60 | 2.06 | 1.03 |
Note. aIFAT; Indirect fluorescent antibody test
 bAAPI; Annual antibody positive index determined by IFAT
 cAPI; Annual parasite index of 2005 and 2006
 * Significant between AAPI and positive rate of IFAT was analyzed by Two-way ANOVA (P = 0.5699).
 # Significant between API and positive rate of IFAT was analyzed by Two-way ANOVA (2005; P = 0.9076, 2006; P = 0.5674).
 & Significant between API and AAPI was analyzed by Two-way ANOVA (2005; P = 0.4478, 2006; P = 0.0116).
