# Supplementary material for: Estimating the malaria transmission of Plasmodium vivax based on serodiagnosis
Source: Malar J. 2012 Aug 1;11:257. doi: 10.1186/1475-2875-11-257 (PMC3470937; doi:10.1186/1475-2875-11-257)
Supplement: Additional file 5: — Positive rate of fluorescent antibody responses of sera in Cheorwon surveyed area. [file 1475-2875-11-257-S5.ppt]

## Slide 1
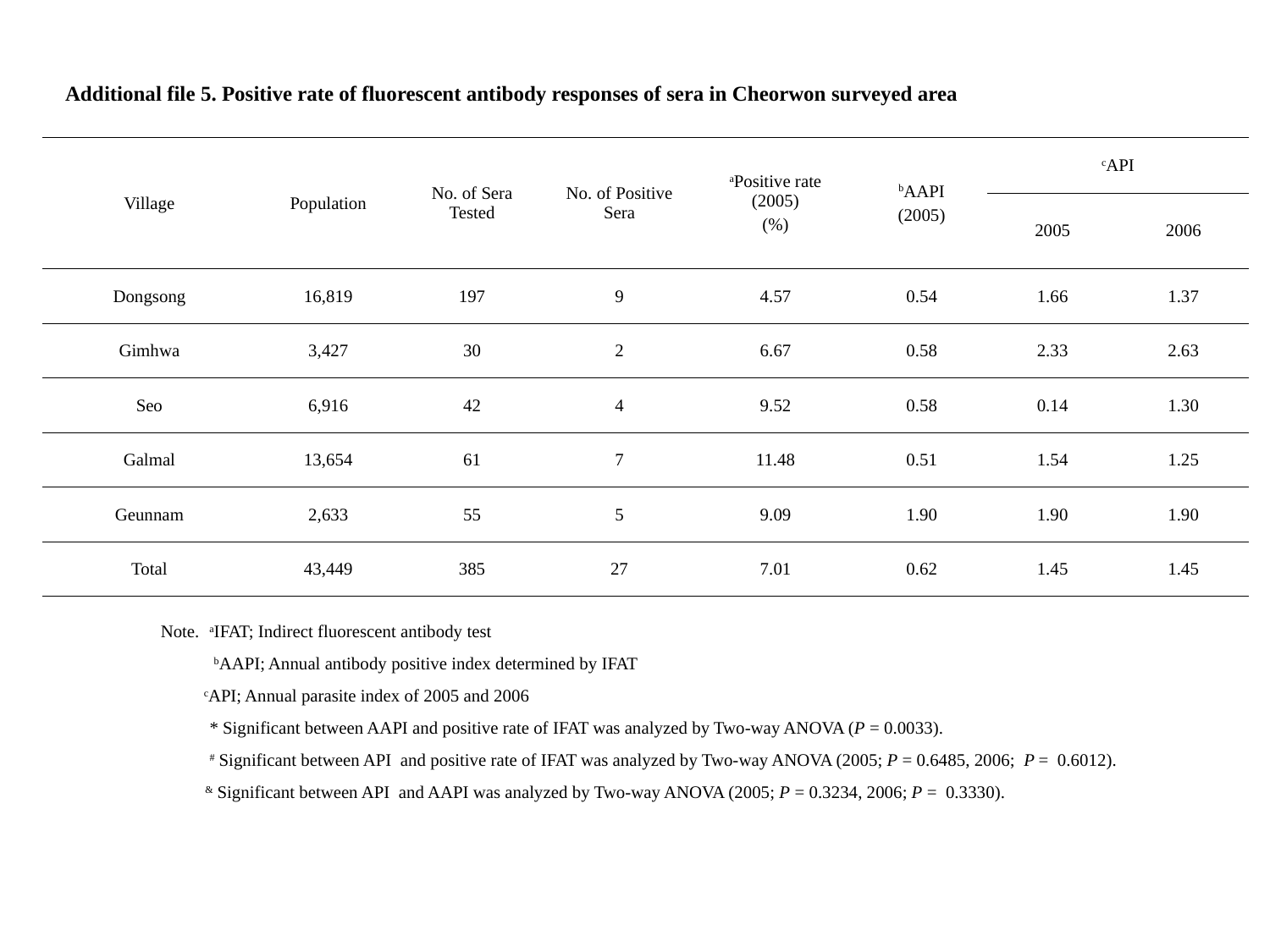

Additional file 5. Positive rate of fluorescent antibody responses of sera in Cheorwon surveyed area
| Village | Population | No. of Sera Tested | No. of Positive Sera | aPositive rate (2005) (%) | bAAPI (2005) | cAPI | |
| --- | --- | --- | --- | --- | --- | --- | --- |
| | | | | | | 2005 | 2006 |
| Dongsong | 16,819 | 197 | 9 | 4.57 | 0.54 | 1.66 | 1.37 |
| Gimhwa | 3,427 | 30 | 2 | 6.67 | 0.58 | 2.33 | 2.63 |
| Seo | 6,916 | 42 | 4 | 9.52 | 0.58 | 0.14 | 1.30 |
| Galmal | 13,654 | 61 | 7 | 11.48 | 0.51 | 1.54 | 1.25 |
| Geunnam | 2,633 | 55 | 5 | 9.09 | 1.90 | 1.90 | 1.90 |
| Total | 43,449 | 385 | 27 | 7.01 | 0.62 | 1.45 | 1.45 |
Note. aIFAT; Indirect fluorescent antibody test
 bAAPI; Annual antibody positive index determined by IFAT
 cAPI; Annual parasite index of 2005 and 2006
 * Significant between AAPI and positive rate of IFAT was analyzed by Two-way ANOVA (P = 0.0033).
 # Significant between API and positive rate of IFAT was analyzed by Two-way ANOVA (2005; P = 0.6485, 2006; P = 0.6012).
 & Significant between API and AAPI was analyzed by Two-way ANOVA (2005; P = 0.3234, 2006; P = 0.3330).
