# Supplementary material for: Estimating the malaria transmission of Plasmodium vivax based on serodiagnosis
Source: Malar J. 2012 Aug 1;11:257. doi: 10.1186/1475-2875-11-257 (PMC3470937; doi:10.1186/1475-2875-11-257)
Supplement: Additional file 6: — Positive rate of fluorescent antibody responses of sera in Goseong surveyed area. [file 1475-2875-11-257-S6.ppt]

## Slide 1
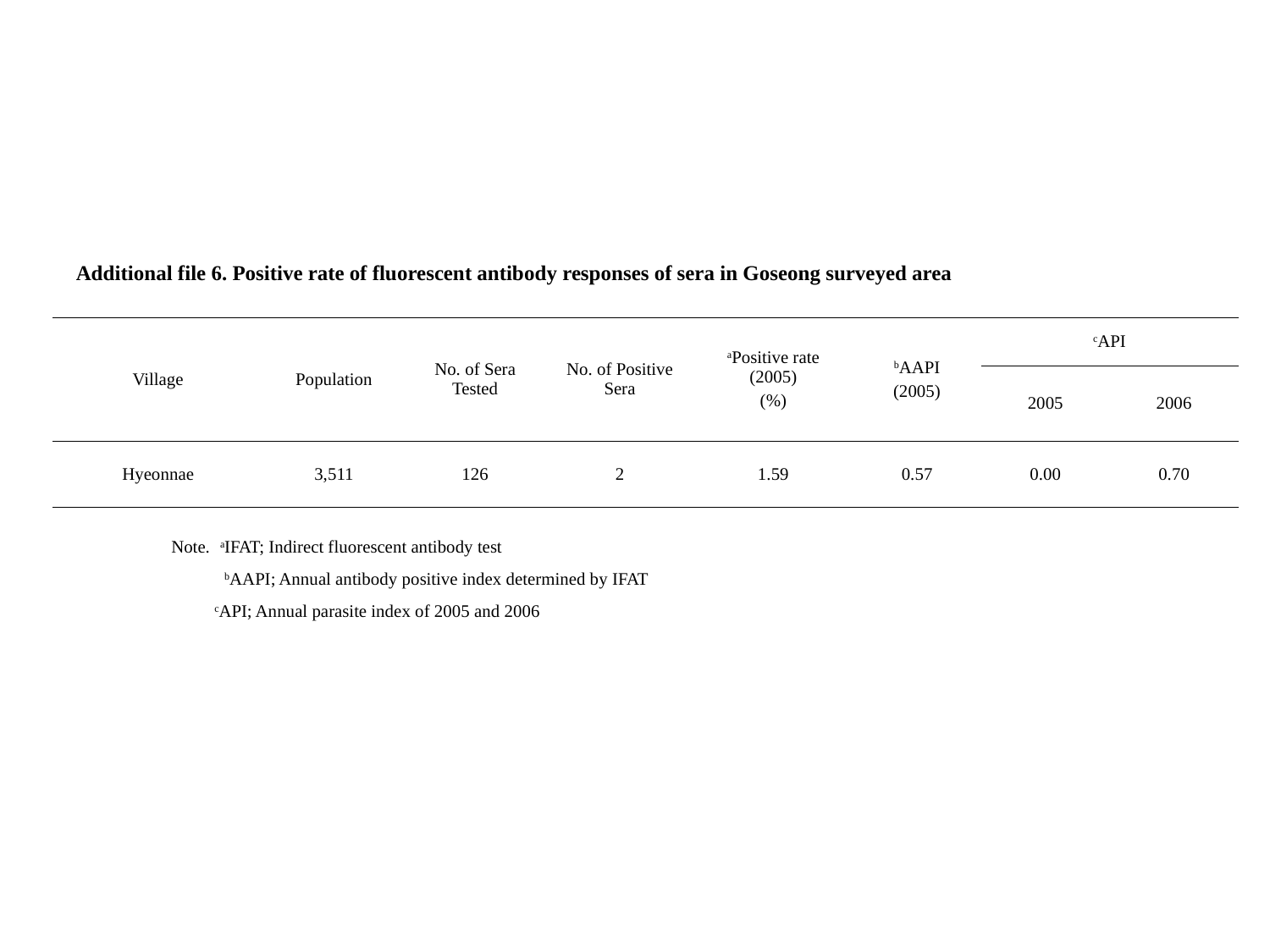

Additional file 6. Positive rate of fluorescent antibody responses of sera in Goseong surveyed area
| Village | Population | No. of Sera Tested | No. of Positive Sera | aPositive rate (2005) (%) | bAAPI (2005) | cAPI | |
| --- | --- | --- | --- | --- | --- | --- | --- |
| | | | | | | 2005 | 2006 |
| Hyeonnae | 3,511 | 126 | 2 | 1.59 | 0.57 | 0.00 | 0.70 |
Note. aIFAT; Indirect fluorescent antibody test
 bAAPI; Annual antibody positive index determined by IFAT
 cAPI; Annual parasite index of 2005 and 2006
